# Evaluation of an interdisciplinary electronic consultation service between general practitioners and medical specialists in The Netherlands: a prospective cohort study

**DOI:** 10.1093/fampra/cmag004

**Published:** 2026-03-05

**Authors:** Ken M M Peeters, Dennis M J Muris, Juliette Klein Hesselink, Kirsten R A Laeven, Tessa Schotman, Guus G de Vries, Roel Hendrickx, Ladbon Khajeh, Jan Stoot, Paul Bergmans, Mariëlle Krekels, Jochen W L Cals

**Affiliations:** Department of Family Medicine, Care and Public Health Research Institute (CAPHRI), Maastricht University, Maastricht 6229 HA, The Netherlands; Zuyderland Medical Centre, Sittard, The Netherlands; Medical Coordination Centre Omnes, Sittard, The Netherlands; Department of Family Medicine, Care and Public Health Research Institute (CAPHRI), Maastricht University, Maastricht 6229 HA, The Netherlands; GGD South-Limburg, Heerlen, The Netherlands; Department of Family Medicine, Care and Public Health Research Institute (CAPHRI), Maastricht University, Maastricht 6229 HA, The Netherlands; Department of Family Medicine, Care and Public Health Research Institute (CAPHRI), Maastricht University, Maastricht 6229 HA, The Netherlands; Department of Family Medicine, Care and Public Health Research Institute (CAPHRI), Maastricht University, Maastricht 6229 HA, The Netherlands; Department of Family Medicine, Care and Public Health Research Institute (CAPHRI), Maastricht University, Maastricht 6229 HA, The Netherlands; Zuyderland Medical Centre, Sittard, The Netherlands; Zuyderland Medical Centre, Sittard, The Netherlands; Zuyderland Medical Centre, Sittard, The Netherlands; Medical Coordination Centre Omnes, Sittard, The Netherlands; Zuyderland Medical Centre, Sittard, The Netherlands; Department of Family Medicine, Care and Public Health Research Institute (CAPHRI), Maastricht University, Maastricht 6229 HA, The Netherlands

**Keywords:** e-consultation, e-consult, electronic consultation, digital consultation, digital interdisciplinary consultation, interdisciplinary consultation

## Abstract

**Background:**

General practitioners (GPs) frequently encounter complex cases that require specialist input. Traditionally, this support is sought via telephone consultations, which are often constrained by time and availability, or through initiating a hospital referral. Interdisciplinary electronic consultations enable timely specialist advice while possibly reducing unnecessary referrals. Though prior research has shown promising outcomes for e-consultations, evidence remains limited, particularly from healthcare systems where GPs act as gatekeepers, such as in The Netherlands. It is also unclear whether similar benefits apply across other hospital departments.

**Objective:**

This study aimed to evaluate how GPs in a Dutch healthcare setting used e-consultations across multiple hospital departments, focusing on both the content of the consultations and their impact on referral decisions.

**Methods:**

We analyzed 2183 e-consultations submitted to Zuyderland Medical Centre between 2019 and 2022, with at least 24 months of data collected per department. Consultations were categorized by topic, and referral outcomes were assessed—specifically avoided versus additional referrals prompted by the e-consult.

**Results:**

E-consultations were used across all departments and addressed a wide range of clinical questions, most commonly related to diagnosis, diagnostic testing, and pharmacological treatment. In surgical departments, questions more often concerned general advice. Overall, 36.8% were avoided, while 11.9% additional referrals followed the e-consult. Avoided referral rates ranged from 32.6% in Pediatrics to 49.0% in Urology.

**Conclusion:**

E-consultations support GPs in managing clinical uncertainty and may reduce unnecessary referrals, particularly when tailored to departmental contexts.

Key messages:

- E-consults may improve access to specialist input and reduce referrals.
- There is limited research from healthcare systems where GP has a gatekeeping role.
- We observed wide use of e-consults across all specialties and question types.
- Many e-consults avoided referrals; few resulted in additional referrals.
- Demonstrates e-consultation's value as a tool for supporting GPs.

## Introduction

### Background

In daily practice, general practitioners (GPs) often encounter patients with complex and diverse complaints, which pose challenges in diagnosis, medication management, and treatment planning. Collaboration with medical specialists can help GPs reduce uncertainty [1]. In the Dutch healthcare system GPs act as gatekeepers, managing patient access to specialist care and handling complex cases as the first point of contact. This structure aims to control healthcare costs and streamline patient care. Medical specialists, with their advanced knowledge and expertise in specific medical fields, provide valuable insights and support to GPs facing difficult cases [2]. Currently, interdisciplinary consultations between GPs and specialists are often conducted by phone, which presents several challenges: calling at an inconvenient time, difficulty reaching the most qualified specialist, and the fact that the specialist may not have access to complete patient information during the call [3–5]. E-consultation offers a solution by enabling asynchronous, digital, interdisciplinary communication between GPs and specialists. E-consultation could support the gatekeeper role of GPs by providing GPs with timely specialist input without requiring immediate in-person referrals.

In interviews conducted by our group, both GPs and medical specialists expressed positive views on e-consultations, as they improve access to care, increase efficiency, and have educational value for GPs [2]. Despite this potential, research on e-consultation remains limited, particularly in healthcare systems with a strong gatekeeper role for GPs, such as in The Netherlands. Our systematic review on this topic showed that most research on e-consultation has been conducted in North America, with a wide variation in outcomes [5]. Investigating e-consultation in this context is crucial, as it may reduce unnecessary referrals, enhance GP decision-making, and alleviate pressure on hospital resources—insights that could be valuable for other gatekeeper-based healthcare systems. Healthcare costs in Europe are rising due to an ageing population, chronic illnesses, and medical advancements [6]. Hospital care plays a significant role in these costs, and some of this care could potentially be avoided through effective digital interdisciplinary consultation. There is a significant staffing shortage in healthcare, making it essential to avoid unnecessary care wherever possible.

A previous study in The Netherlands by Muris *et al*. [7] examined the use of e-consultation at the department of Internal Medicine showing that 48.7% of all e-consultations led to an avoided referral. E-consultations were used for a wide range of questions across all areas of Internal Medicine. While e-consultation has shown potential in departments like Internal Medicine, where the approach is reflective and diagnostic questions are often complex, it remains uncertain if similar benefits would apply across various other departments. For example, surgical specialties might require physical examination and direct interventions, potentially limiting the efficacy of e-consultation.

### Objective

To further develop and scale e-consultation, it is essential to gain insight into department-specific usage which can help optimize the current e-consultation system. This study aimed to examine the usage of e-consultations across diverse departments and to assess their impact on referral rates, with a focus on identifying patterns in avoided versus additional referrals.

## Materials and methods

### Study design, setting, and population

We conducted a prospective cohort study between April 2019 and March 2023. The Zuyderland MC's Medical Ethical Research Committee (14-N-69) approved this study. We included patients that were 18 years or older (apart from the department of Pediatrics) for whom the GP submitted an e-consultation to one of the following departments within the Zuyderland Medical Centre: Neurology, Pulmonology, Pediatrics, General Surgery, Orthopedics, Urology. We excluded patients already under treatment by that specific medical specialty, patients in acute emergency situations, and e-consultations with incomplete data. These departments were selected because they represent specialties that receive a high volume of e-consultations and provide a balanced mix of medical and surgical specialties.

### The e-consultation process

In the southern region of The Netherlands, GPs, including locum doctors and medical trainees, have access to e-consultations with medical specialists in participating departments. This area had approximately 190 000 inhabitants in 2023. This e-consultation was facilitated by MCC Omnes in 2016 and is the first large-scale e-consultation project in The Netherlands. The e-consultation is facilitated through the secure referral application “ZorgDomein.” The process of submitting an e-consultation involves the GP informing the patient, submitting the e-consultation containing a clinical question and patient information, and having an assistant schedule the consultation in the specialist's calendar. Within five working days, the specialist responds to the GP via an electronic letter. There is currently no option for follow-up questions. A more detailed description of the process can be found in Peeters *et al*. [2].

### Data acquisition and analysis

Multiple researchers extracted data from electronic medical records. This included characteristics of the patient, GP, and medical specialist. They also recorded the diagnostics performed by the GP, including any additional laboratory tests or medical imaging. Furthermore, they analyzed the subject and clinical question of the e-consultation, the response time, and the type of advice given by the medical specialist. Finally, they examined whether a referral was recommended and whether it actually took place within six months after the e-consultation. To identify patients who had been referred after e-consultation or patients who were already under treatment we analyzed the healthcare products; the healthcare activities registered in the hospital's administrative system for reimbursement purposes. Consecutive e-consultations were collected over different periods between April 2019 and March 2023 across various hospital departments by a group of researchers (K.P., G.V., K.L., J.K.H., and T.S.), with a minimum of 24 months of data collected per department. The data were anonymized and the type of questions, advice, and topic of e-consultation were classified using a previously created classification system. This classification system was developed for each department, in cooperation with a medical specialist (P.P., L.K., R.H., and J.S.). In case of doubt, a consensus meeting was held in the research team which consisted of a GP and two other researchers (J.C., D.M., and K.P.) to find an agreement. We performed descriptive statistics using R (version 4.4.2).

When submitting an e-consultation in ZorgDomein, a GP was obligated to answer the following question: “Would you have referred the patient if e-consultation would not have been possible?” Table 1 shows the four possible referral scenarios following the e-consultation.

**Table 1:** Four possible referral scenarios following e-consultation.

GP intention to refer?	Patient actually referred?		Total
	Yes	No	
Yes	Still needed n (%)	Avoided referrals n (%)	Total number where GP intended to refer n (%)
No	Extra referrals n (%)	Still not needed n (%)	Total number where GP did not intend to refer n (%)
Total	Total referred n (%)	Total not referred n (%)	Total number of patients n

GP, general practitioner.

## Results

### Characteristics of patients, general practitioners, and medical specialists

In total, 2585 e-consultations submitted by 179 GPs were collected. At the time of analysis, there were 136 established GPs in this region. We excluded 402 e-consultations: patients under 18 years old, with the exception of Pediatrics (114), patients already under treatment by a medical specialist (104), technical errors with retrieving the e-consultation (77), no response by the medical specialist (72), double requests (23), or e-consultations sent to the wrong department (12). After exclusion, 2183 e-consultations were included for analysis. Table 2 shows the characteristics of the included e-consultations. The median number of e-consultations per month ranged from 4 to 19 e-consultations across the included departments. The median response time was 1 working day, ranging from 0 to 46 working days.

**Table 2:** Characteristics of patients for which GP initiated an e-consultation.

	Total *n* (%)^a^
**E-consultations**	**2183**
Sex	
Female	1238 (56.7%)
Male	945 (43.3%)
Age in years; mean (SD)	53.3 (23.8)
Answered within 5 workdays	2105 (96.4%)
**Departments**	
Neurology	744
Pulmonology	352
Pediatrics	236
General surgery	175
Orthopedics	480
Urology	196
**E-consultations per month**	**39** (**1–104)**
Neurology; median (range)	15 (4–25)
Pulmonology; median (range)	7 (1–16)
Pediatrics; median (range)	7.5 (3–18)
General surgery; median (range)	4 (1–12)
Orthopedics; median (range)	19 (1–37)
Urology; median (range)	6 (1–23)

^a^Unless stated differently.

### Contents of e-consultation

Table 3 shows the type of questions asked by GPs. The most frequent type of question concerned the requirement for a referral for a face-to-face visit. Other frequently occurring questions concerned a diagnosis, general advice, diagnostic test(s), or drug treatment. Under “general advice,” we classified everything that did not fit into other categories. GPs utilized the e-consultations for a broad range of questions across all subspecialties of all departments (Supplementary Appendix S1). Notably, the category “Other” appeared among the five most common topics across various departments and was even the most frequent topic for two departments.

**Table 3:** The type of questions asked by general practitioners in the e-consultation, per department.

Question n (%)	Total (n = 2457)	Neurology (n = 958)	Pulmonology (n = 440)	Pediatrics (n = 335)	General surgery (*n* = 175)	Orthopedics (*n* = 347)	Urology (*n* = 201)
**Requirement for referral for face-to-face visit**	530 (21.6%)	210 (21.9%)	85 (19.3%)	60 (17.9%)	37 (21.1%)	94 (27.1%)	44 (21.9%)
**General advice**	498 (20.3%)	169 (17.6%)	93 (21.1%)	76 (22.6%)	35 (20.0%)	82 (23.6%)	43 (21.4%)
**Drug treatment**	445 (18.1%)	234 (24.4%)	74 (16.8%)	32 (9.5%)	20 (11.4%)	46 (13.3%)	39 (19.4%)
**Diagnostic test(s)**	438 (17.8%)	123 (12.8%)	109 (24.7%)	79 (23.5%)	34 (19.4%)	52 (15.0%)	41 (20.4%)
**(Differential) diagnosis**	377 (15.3%)	174 (18.2%)	55 (12.5%)	46 (13.7%)	27 (15.4%)	44 (12.7%)	31 (15.4%)
**Other people involved**	75 (3.1%)	13 (1.4%)	2 (0.5%)	19 (5.7%)	15 (8.6%)	25 (7.2%)	1 (0.5%)
**Prognosis**	61 (2.5%)	27 (2.8%)	4 (0.9%)	21 (6.3%)	4 (2.3%)	4 (1.2%)	1 (0.5%)
**Guidelines**	22 (0.9%)	4 (0.4%)	17 (3.9%)	1 (0.3%)	0 (0%)	0 (0%)	0 (0%)
**Heredity**	11 (0.5%)	4 (0.4%)	1 (0.2%)	2 (0.6%)	3 (1.7%)	0 (0%)	1 (0.5%)

The number of questions does not equal the number of e-consultations, since an e-consultation can contain multiple questions.

Table 4 shows the types of advice given by medical specialists. The most frequent type of advice concerned the requirement for a referral for a face-to-face visit. Other frequently occurring types of advice concerned a diagnosis, drug treatment, or diagnostic test(s). The number of provided advice exceeded the number of questions asked, as advice was often given in the form of a step-by-step plan. For example, a question seeking general advice, e.g. “What should I do?” was followed by recommendations concerning a potential diagnosis, possible medication, and, if this did not work, advice for a referral. Notably, fewer pieces of advice on diagnosis were given by the surgical departments, and more general advice was provided.

**Table 4:** The type of advice given by medical specialists in the e-consultation, per department.

Advice *n* (%)	Total (*n* = 3335)	Neurology (*n* = 1292)	Pulmonology (*n* = 661)	Pediatrics (*n* = 554)	General surgery (*n* = 216)	Orthopedics (*n* = 342)	Urology (*n* = 270)
**Requirement for referral for face-to-face visit**	938 (28.1%)	431 (33.4%)	165 (25.0%)	111 (20.0%)	70 (32.4%)	93 (27.1%)	68 (25.2%)
**(Differential) diagnosis**	634 (19.0%)	325 (28.3%)	115 (17.4%)	124 (22.4%)	19 (8.8%)	28 (8.2%)	23 (8.5%)
**Drug treatment**	584 (17.5%)	232 (18.0%)	135 (20.4%)	80 (14.4%)	17 (7.8%)	67 (20.0%)	53 (19.6%)
**Diagnostic test(s)**	452 (13.6%)	72 (5.6%)	157 (23.8%)	63 (11.4%)	33 (15.3%)	60 (17.5%)	67 (24.8%)
**General advice**	383 (11.5%)	155 (12.0%)	50 (7.6%)	27 (4.9%)	33 (15.3%)	70 (20.5%)	48 (17.8%)
**Prognosis**	149 (4.5%)	41 (3.2%)	9 (1.4%)	84 (15.2%)	7 (3.2%)	3 (0.8%)	5 (1.9%)
**Other people involved**	134 (4.0%)	18 (1.4%)	3 (0.4%)	56 (10.1%)	30 (13.9%)	21 (6.1%)	6 (2.2%)
**Guidelines**	49 (1.5%)	14 (1.1%)	27 (4.1%)	8 (1.4%)	0 (0%)	0 (0%)	0 (0%)
**Heredity**	12 (0.4%)	4 (0.3%)	0 (0%)	1 (0.2%)	7 (3.2%)	0 (0%)	0 (0%)

The number of advice does not equal the number of e-consultations, since an e-consultation can contain multiple advices.

### Follow-up after e-consultation

Across departments, 41.1% of e-consultations resulted in a referral to an outpatient clinic within 6 months. Without the availability of e-consultation, GPs indicated they would have referred an average of 66.5% of these patients. When referral advice was provided, GPs followed this recommendation in 56.9% of cases. The median wait time, the time between the e-consult by the GP and the outpatient department visit by the patient, was 31 days (range 1–99 days). Table 5 shows that across departments, an average of 36.8% referrals were avoided by the implementation of e-consultation. An additional 11.9% referrals followed e-consultation, where at first the GP did not consider referral. The percentage of avoided referrals varied from 32.6% for the department of Pediatrics to 49.0% for the department of Urology. The percentage of extra referrals varied from 8.2% for the department of Urology to 15.6% for the department of Pulmonology.

**Table 5:** Referral scenarios within 6 months after submitting e-consultation, per department.

	Still needed; *n* (%)	Avoided referrals; *n* (%)	Extra referrals; *n* (%)	Still not needed; *n* (%)	Total; *n*
**Neurology**	236 (31.7%)	276 (37.1%)	92 (12.4%)	140 (18.8%)	744
**Pulmonology**	72 (20.5%)	123 (34.9%)	55 (15.6%)	102 (29.0%)	352
**Pediatrics**	66 (28.0%)	77 (32.6%)	26 (11.0%)	67 (28.4%)	236
**General surgery**	59 (33.7%)	60 (34.3%)	23 (13.1%)	33 (18.9%)	175
**Orthopedics**	190 (39.6%)	171 (35.6%)	52 (10.8%)	67 (14.0%)	480
**Urology**	43 (21.9%)	96 (49.0%)	16 (8.2%)	41 (20.9%)	196
**Total^a^**	29.2%	37.2%	11.9%	21.7%	

^a^Percentages in the “Total” row represent the unweighted mean percentage across departments, so that each department contributes equally regardless of the number of e-consultations.

## Discussion

### Principal findings

This study evaluated GP use of interdisciplinary e-consultations across multiple hospital departments. We analyzed 2183 e-consultations and found broad utilization of e-consultations across all departments, addressing a wide range of questions and topics. The most common inquiries concerned diagnoses, diagnostic tests, and drug treatment. Surgical specialties, however, focused less on diagnosis and more on general advice. Across all departments, e-consultations led to avoided referrals (36.8%), with only a small percentage resulting in additional referrals (11.9%).

### Comparison with existing literature

Other literature shows that e-consultations are used for a wide range of questions and topics across different countries and hospital departments. However, the reasons for implementation may differ [5]. The Netherlands is a densely populated country, where access to healthcare in general and time to access in particular may be less of an issue compared with parts of North America. Therefore, one could argue that implementing e-consultations to improve healthcare access and prevent unnecessary referrals might be a more prominent motivation in those regions than in The Netherlands, where e-consultations may primarily support GPs. Additionally, in The Netherlands, GPs serve as gatekeepers, playing a crucial role in controlling access to specialist care. This gatekeeper function may lead to lower referral rates and more conservative use of e-consultations compared with healthcare systems where patients can access hospital care directly. Referral rates vary widely, even across studies conducted within the same country [5]. This variation may be due to factors such as the nature of the specialty, the implementation process, the type of health insurance, reimbursement policies, and the workload of GPs and specialists.

When comparing the departments analyzed in this study with the previous research conducted at the department of Internal Medicine [7], we observe that the percentages of avoided referrals and “still not needed” referrals are lower compared with the department of Internal Medicine (48.7% and 32.5% respectively), while the percentages of extra referrals and “still needed” referrals are higher compared with the department of Internal Medicine (4.9% and 13.7%, respectively). An exception is the department of Urology, which also shows a high number of avoided referrals (49.0%). The higher rates of avoided and “still not needed” referrals could be from the nature of conditions treated, which often include chronic or general medical issues that can be managed effectively through remote advice, such as medication adjustments or lifestyle guidance. Additionally, the department of Internal Medicine generally faces fewer cases requiring immediate physical examination, compared with surgical departments for example, making remote consultations more practical. Finally, the department may use conservative management protocols that support monitoring and follow-up without the need for immediate in-person visits, further contributing to the effectiveness of e-consultations in avoiding unnecessary referrals.

### Strengths and limitations

This study utilizes a large number of e-consultations across multiple departments. We chose to only include patients referred to a certain hospital. This led to possible referrals being overlooked that were made to different hospitals or paramedical centers, and therefore a possible underestimation of the actual referrals that were made. It is expected that this is of minimal influence since this hospital is the only hospital in the area and therefore the number of patients being referred to other hospitals will be minimal. Furthermore, GPs in the study region order diagnostic testing, both laboratory and medical imaging, in close collaboration with the hospital, and hence diagnostic results can be viewed by both GPs and specialists. This is a crucial component for the current implementation of e-consultation since relevant diagnostics performed by GPs could be reviewed at all times by specialists giving the advice and vice versa. Therefore, a smooth collaboration between primary and secondary care can be ensured in this study. This study was conducted in a period during which the COVID-19 pandemic started, therefore possibly influencing the number of referrals to the hospital or people visiting their GP. During the COVID-19 pandemic, hospitals experienced significant strain, which may have led to delays or barriers in patient referrals following e-consultation [8, 9]. However, data indicate that despite the decrease in hospital referrals during the COVID-19 period, the number of e-consultations remained unchanged, and we did not encounter any comments in the responses from medical specialists suggesting that referrals should be avoided due to hospital strain.

### Implications for clinicians and policy makers

The analysis of the types of questions and topics frequently addressed in e-consultations is important as these findings can inform best practices for e-consultation use and guidance of education, helping clinicians recognize for which topics e-consultation can be most applicable. Noteworthy in this study was the frequent occurrence of the category other across multiple departments. The classification list used to categorize the topics was developed in collaboration with medical specialists. This may support the idea that e-consultations are often employed in cases of uncertainty, where the issue cannot easily be classified under an existing diagnosis. We found that the e-consultation is relevant for a wide range of questions and topics showing that e-consultations are flexible and adaptable to various clinical scenarios, highlighting their potential value in supporting clinicians across different specialties. E-consultations were used by a large number of GPs (*n* = 179). Locum doctors and medical trainees were also able to request e-consultations, which is why the number of applicants exceeds the number of established GPs (*n* = 136). For surgical departments, there were fewer specific recommendations on diagnoses and there was more general advice. Under general advice, we classified e-consults that did not fit into other categories, including recommendations on possible interventions. The higher amount of general advice could be because questions directed to surgical departments focus less on diagnosis, as is more common at the department of Internal Medicine for example, and more on potential treatment options.

Analyzing referrals is important for policymakers, with particular emphasis on distinguishing between avoided referrals and extra referrals. By differentiating between these types of referrals, we can assess whether the e-consultation service generates increased demand for care—with the availability of care leading to a corresponding increase in its use. In this case, GPs might refer patients to the hospital through e-consultation, even when they would not have otherwise, or request an e-consultation for a patient for whom they would typically not have taken any action. The study shows that the number of extra referrals following e-consultation is low (8.2% to 15.6%), while the number of avoided referrals is much higher (32.6% to 49.0%). These avoided referrals could help control healthcare costs. This is particularly important as there is a major challenge regarding staffing shortages in healthcare in Europe [6]. The demand for healthcare is increasing substantially due to ageing populations, chronic diseases, and medical advancements, with hospital costs accounting for a large portion of these expenses. The e-consultation model could play a role in maintaining healthcare accessibility and affordability. It is important to note that in the categories “referral still needed” and “referral still not needed,” e-consultations lead to a small amount of additional healthcare utilization, specifically the e-consultation itself. However, this does not mean that the e-consultation offers no added value in these cases, as the specialist is better prepared for the patient's hospital visit due to the preliminary question and shared documentation [2]. Extra referrals can also provide added value to healthcare, as they ensure that patients who would normally not be seen by a specialist are given the opportunity to do so. A notable finding is that the specialist's referral advice in e-consultations is followed in 56.9% of cases. However, this can be explained by several factors. In some instances, the advice was merely a suggestion rather than a clear recommendation for referral, meaning that not all suggestions necessarily needed to be followed. Additionally, GPs may take other factors into account, such as patient preferences, evolving conditions, or their own clinical judgment, when making referral decisions. Understanding these considerations could help further enhance the role of e-consultations in optimizing referral practices.

A number of e-consultations were not answered or responded to with delay. Although our data do not allow us to determine the exact reasons, it is likely that many of these cases were already resolved through other communication channels (e.g. by phone). To prevent unanswered or delayed responses, adequate training in formulating and responding to e-consultation questions, as well as strong regional collaboration, is important.

## Conclusion

Interdisciplinary e-consultations are widely used across multiple departments to address a broad range of questions, although differences in usage between departments exist. More than one third of the e-consultations lead to avoided referrals with minimal additional ones. This demonstrates their value as a tool for both supporting GPs in managing uncertainty and knowledge gaps, and in improving healthcare accessibility and affordability, warranting further adoption across departments.

## Supplementary Material

cmag004_Supplementary_Data

## Data Availability

The data underlying this article will be shared on reasonable request to the corresponding author.
