# Supplementary material for: Evaluation of an interdisciplinary electronic consultation service between general practitioners and medical specialists in The Netherlands: a prospective cohort study
Source: Fam Pract. 2026 Mar 5;43(2):cmag004. doi: 10.1093/fampra/cmag004 (PMC13016908; doi:10.1093/fampra/cmag004)
Supplement: cmag004_Supplementary_Data [file cmag004_supplementary_data.pdf]

Appendix 1: The five most frequently mentioned topics in e-consultations, per department.

	1	2	3	4	5
**Neurology; n (%) n = 744**	Other (136; 18.3%)	Cerebrovascular disorders (107; 14.4%)	Headache (105; 14.1%)	Polyneuropathy (87; 11.7%)	Radicular syndrome (81; 10.9%)
**Pulmonology; n (%) n = 352**	Other (94; 26.7%)	Dyspnoea (70; 19.9%)	Coughing (59; 16.8%)	COPD (58; 16.5%)	Asthma (28; 8.0%)
**Pediatrics; n (%) n = 236**	Other (93; 39.4%)	Chronic abdominal pain / obstipation (23; 9.7%)	Other gastro-enterological disorders (18; 7.6%)	Other neurological disorders (15; 6.4%)	Disorders scrotum / testis / penis (14; 5.9%)
**General surgery; n (%) n = 175**	Vascular surgery (49; 28.0%)	Oncological surgery (24; 13.7%)	Trauma (21; 12.0%)	Disorder of the abdominal wall, groin canal or scrotum (20; 11.4%)	Middle GI surgery (18; 10.3%)
**Orthopedics; n (%) n = 480**	Tendon/muscle ruptures (109; 22.7%)	Other (95; 19.8%)	Fractures (83; 17.3%)	Arthrosis (68; 14.2%)	Tendinitis (35; 7.3%)
**Urology; n (%) n = 196**	Urinary tract infections (32; 16.3%)	Elevated PSA or abnormal RT (29; 14.8%)	LUTS (Lower Urinary Tract Symptoms) (21; 10.7%)	Scrotal abnormality (18; 9.2%)	Abnormality penis (15; 7.7%)
